# Prior Tonsillectomy and the Risk of Breast Cancer in Females: A Systematic Review and Meta-analysis

**DOI:** 10.3389/fonc.2022.925596

**Published:** 2022-07-20

**Authors:** Salah Eddine O. Kacimi, Anas Elgenidy, Huzaifa Ahmad Cheema, Mounir Ould Setti, Atulya Aman Khosla, Amira Yasmine Benmelouka, Mohammad Aloulou, Kawthar Djebabria, Laila Salah Shamseldin, Omar Riffi, Nabil Smain Mesli, Hanane Z. Sekkal, Ahmed M. Afifi, Jaffer Shah, Sherief Ghozy

**Affiliations:** ^1^ Faculty of Medicine, Abou-Bekr Belkaid University of Tlemcen, Tlemcen, Algeria; ^2^ Faculty of Medicine, Cairo University, Cairo, Egypt; ^3^ Department of Medicine, King Edward Medical University, Lahore, Pakistan; ^4^ Institute of Public Health and Clinical Nutrition, University of Eastern Finland, Kuopio, Finland; ^5^ Global Database Studies, IQVIA, Espoo, Finland; ^6^ Maulana Azad Medical College, New Delhi, India; ^7^ Faculty of Medicine, University of Algiers, Algiers, Algeria; ^8^ Faculty of Medicine, University of Aleppo, Aleppo, Syria; ^9^ Faculty of Medicine, University of Annaba, Annaba, Algeria; ^10^ Faculty of Medicine, Tanta University, Tanta, Egypt; ^11^ Department of Surgery A, University-Hospital Center (CHU) of Tlemcen, Tlemcen, Algeria; ^12^ Division of Gastroenterology, Baylor College of Medicine, Houston, TX, United States; ^13^ New York State Department of Health, Albany, NY, United States; ^14^ Department of Radiology, Mayo Clinic, Rochester, MN, United States; ^15^ Nuffield Department of Primary Care Health Sciences and Department for Continuing Education (EBHC program), Oxford University, Oxford, United Kingdom

**Keywords:** breast, tonsillectomy, meta-analysis, risk, oral infection

## Abstract

**Background:**

Exposure to recurrent infections in childhood was linked to an increased risk of cancer in adulthood. There is also evidence that a history of tonsillectomy, a procedure often performed in children with recurrent infections, is linked to an increased risk of leukemia and Hodgkin lymphoma. Tonsillectomy could be directly associated with cancer risk, or it could be a proxy for another risk factor such as recurrent infections and chronic inflammation. Nevertheless, the role of recurrent childhood infections and tonsillectomy on the one hand, and the risk of breast cancer (BC) in adulthood remain understudied. Our study aims to verify whether a history of tonsillectomy increases the risk of BC in women.

**Methods:**

A systematic review was performed using PubMed, Google Scholar, Scopus, Embase, and Web of Science databases from inception to January 25, 2022, to identify the studies which assessed the association between the history of tonsillectomy and BC in females. Odds ratio (OR) was calculated using the random/fixed-effects models to synthesize the associations between tonsillectomy and BC risk based on heterogeneity.

**Results:**

Eight studies included 2252 patients with breast cancer of which 1151 underwent tonsillectomy and 5314 controls of which 1725 had their tonsils removed. Patients with a history of tonsillectomy showed a higher subsequent risk of developing BC (OR, 1.24; 95% CI: 1.11-1.39) as compared to patients without a history of tonsillectomy. Influence analyses showed that no single study had a significant effect on the overall estimate or the heterogeneity.

**Conclusions:**

Our study revealed that a history of tonsillectomy is associated with an increased risk of breast cancer. These findings underscore the need for frequent follow-ups and screening of tonsillectomy patients to assess for the risk of BC.

## Introduction

Inflammatory processes can increase the risk of cancer development. Tonsillitis is one of the most common presentations of inflammatory diseases, especially in children. Its treatment strategy includes performing a tonsillectomy, which is a routine procedure. The acute complications of this surgery include hemorrhage and infection, but long term, it has also been correlated with a higher risk for neoplastic development (1). Studies have linked it to the development of prostate cancer (2), Hodgkin’s lymphoma (3), and leukemia (4, 5).

Two theories have been proposed in the literature that might explain the risk between tonsillectomies and the development of cancer. The first is that the immune function of tonsils is greatest in childhood and that it drastically decreases after adolescence. Therefore, children with tonsillectomies are put at a greater risk for viral infections which subsequently aid in the development of cancer (3). However, in recent years, a meta-analysis conducted by Bitar et al. found that tonsillectomies do not result in negative immunological sequelae (6). The second, and more plausible theory is that those individuals who develop cancer, have not only predisposing factors but an altered immune function too. This may have made them more susceptible to inflammatory conditions in childhood, like tonsillitis, leading them to have a tonsillectomy (3).

The main aim of our study was to conduct the first comprehensive critical review and meta-analysis of observational studies to ascertain the risk of cancer in people with a reported history of tonsillectomy.

## Methods

The study was performed according to the Preferred Reporting Items for Systematic Reviews and Meta-analyses (PRISMA) guidelines (7). The University of Tlemcen institutional review board determined that approval was not required for this study design.

### Search Strategy and Eligibility Criteria

Three electronic databases (PubMed, Google Scholar, Scopus, Embase, and Web of Science) were searched from inception to January 25, 2022, for relevant studies. The details of the search strategy for each database are presented in **Appendix 4**. The searches were limited to human studies and were performed for all languages and study types. Additional studies were identified by 2 independent investigators through manually searching conference abstracts, clinical trial databases, and reference lists.

All included studies had to meet the following eligibility criteria: cohort or case-control study design; at least 1 study group of patients with tonsillectomy; and a comparison group involving patients without tonsillectomy or the general population. Included studies were also required to investigate breast cancer occurrence, incidence, or prevalence of cancer within this group of patients. Studies investigating only a pediatric population were excluded to minimize age-related bias. For overlapping studies from the same cohort (eg, studies based on the same database in the same period or follow-ups of older studies), the latest and most appropriate outcomes were selected by the consensus of all the investigators.

### Study Selection and Data Extraction

Two investigators independently screened the titles and abstracts of all the articles using the predefined inclusion criteria. The full-text articles were examined independently by all investigators to determine whether they met the inclusion criteria. Furthermore, the same authors independently extracted data using a data extraction form. The final inclusion of each article was determined by all investigators’ evaluation discussions. References and data for each included study were carefully cross-checked to ensure that no overlapping data were present and to maintain the integrity of the meta-analysis.

### Critical Appraisal Tool and Risk of Bias Assessment

To assess the risk of bias in the included cohort and case-control studies, the Newcastle-Ottawa Scale tool was used. Using the tool, each study was judged on 8 items in 3 categories, including the selection of the study groups, the comparability of the groups, and the ascertainment of the exposure of interest for case-control studies or the outcome of interest for cohort studies. Studies that received 7, 8, or 9 of a possible 9 points were regarded as high quality, whereas studies that received 4, 5, or 7 were regarded as fair quality (high risk of bias), and those that received 3 or less were regarded as low quality (very high risk of bias) (8).

### Data Analysis

Our meta-analysis was performed using the “meta” package of R software version 4.1.0 (9). We used the inverse variance models for the analyses. I-squared scores > 50% is considered substantial heterogeneity. A *P*-value of less than 0.05 indicates statistically significant results.

We performed subgroup analyses using fixed-effect models, and sensitivity (influence) analysis to show the effect of every single study on the overall effect and heterogeneity.

## Results

### Study Selection

**Figure 1:**
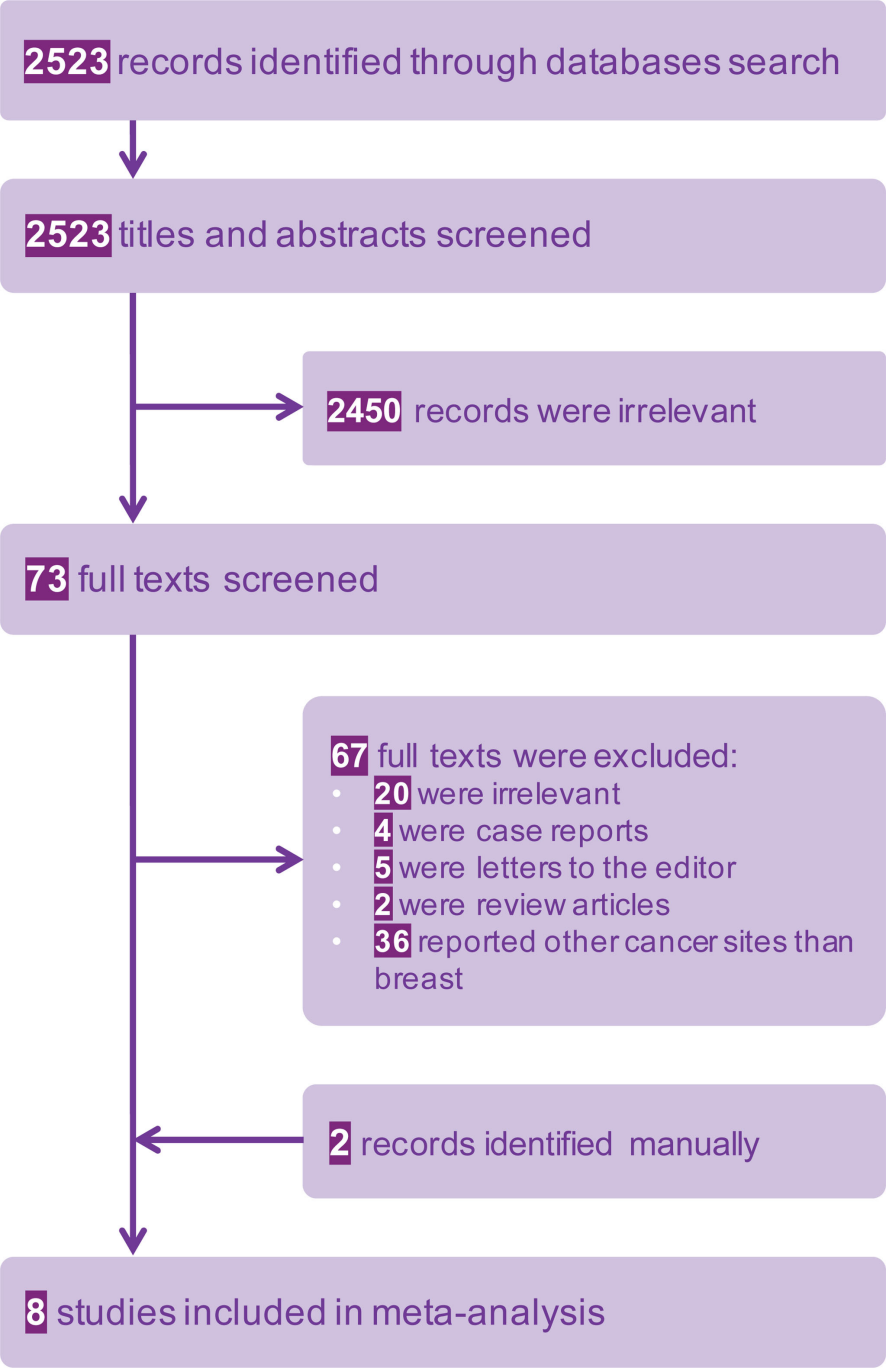
The Prisma flow diagram of the study.

The systematic search identified 2523 potentially relevant studies. After initial review by title and abstracts, 2450 articles were excluded, leaving 73 to be reviewed in full text. Eight studies ultimately met our prespecified criteria and were included in our analysis. The detailed study selection process is depicted in a PRISMA flowchart in **Figure 1**.

### Study Characteristics

Together, these eight studies included 2252 patients with breast cancer of which 1151 underwent tonsillectomy and 5314 controls of which 1725 had their tonsils removed. Seven were case-control studies (10–16) and one was a retrospective cohort study (17). No randomized trials were found. The number of participants in the included studies ranged from 174 to 2200. Four studies were performed in the USA (10, 11, 13, 15) and one each in Scotland (12), Canada (14), Taiwan (17) and Greece (10) (**Table 1**)

**Table 1:** Study characteristics.

Study	Country	Study design	No. of patients with breast cancer	No. of controls	No. of patients with breast cancer with tonsils removed	No. of controls with tonsils removed	Risk measurement	Risk of bias
Brasky et al. (16)	USA	Case control	736	801	389	380	Adj OR	High quality
Gross et al. (11)	USA	Case control	110	200	22	46	NR	Very high risk
Howie et al. (12)	Scotland	Case control	149	478	54	117	NR	High risk
Kessler et al. (13)	USA	Case control	89	85	29	20	NR	Very high risk
Lubin et al. (14)	Canada	Case control	558	824	286	384	Adj OR	High risk
Sun et al. (17)	Taiwan	R. Cohort	440^a^	1760^b^	7	14^c^	IRR	High quality
Yasui et al. (15)	USA	Case control	537	492	362	314	Adj OR	High quality
Cassimos et al. (10)	Greece	Case control	52	255	2	31	NR	Very high risk

a, number of patients with tonsillectomy; b, number of patients without tonsillectomy; c, number of patients with breast cancer without tonsils removed.Adj OR, Adjusted Odds Ratio; IRR, Incidence Rate Ratio; NR, Not Reported; R. Cohort, Retrospective Cohort.

### Risk of Bias in Studies

Three studies were of high quality (15–17) while the rest of the studies had a high risk or very high risk of bias (10–14). Most of the studies did not match the cases and controls or adjust the potential confounders and did not enroll all eligible cases with the outcome of interest over a defined period, all cases in a defined catchment area, or did not include a random sample. The details of the quality assessment are summarized in **Appendix 1**.

#### Synthesis of Data

**Figure 2:**
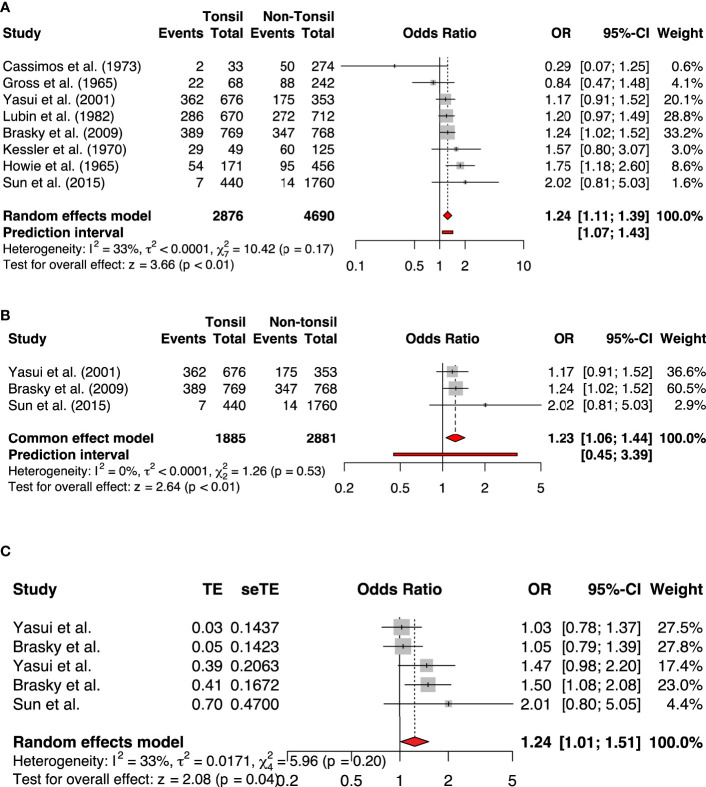
The forest plots of **(A)** All studies included (Unadjusted analysis) **(B)** Only high-quality studies included based on NOS (Unadjusted analysis) **(C)** Adjusted analysis.

Our meta-analysis included eight studies comprising 2876 participants with a history of tonsillectomy and 4690 without a history of tonsillectomy. It revealed a statistically significantly increased risk of breast cancer in the group with a history of tonsillectomy as compared to the group without a history of tonsillectomy (OR, 1.24; 95% CI: 1.11-1.39). The heterogeneity among the studies was of an acceptable level (I^2^ = 33%; *P* = .17; **Figure 2**). We also pooled the data using adjusted effect sizes where available from the included studies. The results were consistent with an increased risk of breast cancer seen in the tonsillectomy group (OR, 1.24; 95% CI: 1.01-1.51, I^2 =^ 33%).

### Investigation of Heterogeneity:

#### Subgroup Analysis

**Figure 3:**
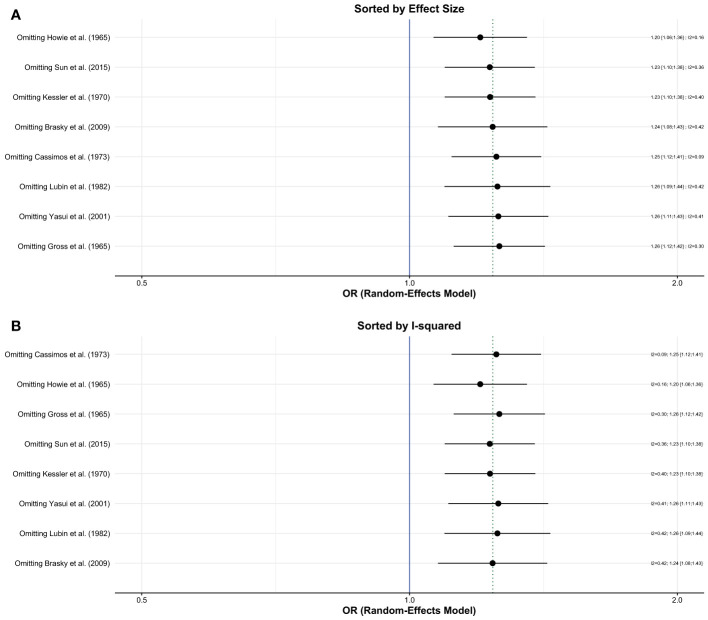
Influence (leave-one out sensitivity) analysis; **(A)** Sorted by effect size **(B)** Sorted by I^2^.

We performed subgroup analyses by menopausal status, year, study design, country, continent, sample size, and quality of studies. Premenopausal women had higher risk of developing breast cancer (OR, 1.71; 95% CI: 1.36-2.15, , I2= 0%). All analyses showed an increased risk of breast cancer in the group with a history of tonsillectomy as compared to the group without a history of tonsillectomy. However, the test for subgroup differences was not significant for any of the comparisons, as shown in **Table 2** and **Figure 3** and figures in **Appendix 3**.

**Table 2:** Results of the subgroup analysis.

Subgroup	All			US			Non-US		
	Studies	OR (95% CI)	I^2^, *p*-value	Studies	OR (95% CI)	I^2^, *p*-value	Studies	OR (95% CI)	I^2^, *p*-value
Age at diagnosis			NA*			NA*			NA*
Premenopausal	2	1.71 (1.36; 2.15)	0%, 0.49	NA	NA	NA	NA	NA	NA
Postmenopausal	2	1.30 (1.05; 1.60)	86%, < 0.01	NA	NA	NA	NA	NA	NA
Study design			0.29*			0.51*			0.34*
Case control	7	1.23 (1.10; 1.38)	36%, 0.16	4	1.20 (1.03; 1.39)	0%, 0.51	3	1.28 (1.06; 1.55)	70%, 0.03
Cohort	1	2.02 (0.81; 5.03)	NA	0	NA	NA	1	2.02 (0.81; 5.03)	NA
Year			0.91*			0.65*			0.34*
> 2000	3	1.23 (1.06; 1.44)	0%, 0.53	2	1.22 (1.04; 1.42)	0%, 0.73	1	2.02 (0.81; 5.03)	NA
< 2000	5	1.25 (1.05; 1.48)	56%, 0.06	2	1.09 (0.71; 1.69)	49%, 0.16	3	1.28 (1.06; 1.55)	70%, 0.03
Continent			0.26*			NA*			0.33*
America	5	1.20 (1.06; 1.36)	0%, 0.68	4	1.20 (1.03; 1.39)	0%, 0.51	1	1.20 (0.97; 1.49)	NA
Europe	2	1.55 (1.06; 2.27)	82%, 0.02	NA	NA	NA	2	1.55 (1.06; 2.27)	82%, 0.02
Asia	1	2.02 (0.81; 5.03)	NA	NA	NA	NA	1	2.02 (0.81; 5.03)	NA
Country			0.49*			NA*			NA*
US	4	1.20 (1.03; 1.39)	0%, 0.51	4	1.20 (1.03; 1.39)	0%, 0.51	NA	NA	NA
Non-US	4	1.30 (1.09; 1.57)	61%, 0.05	NA	NA	NA	4	1.30 (1.09; 1.57)	61%, 0.05
Population			0.99*			0.62*			0.31*
> 1000	3	1.24 (1.07; 1.43)	0%, 0.56	1	1.24 (1.02; 1.52)	NA	2	1.55 (1.06; 2.27)	82%, 0.02
< 1000	5	1.24 (1.03; 1.50)	57%, 0.05	3	1.15 (0.92; 1.44)	2%, 0.36	2	1.24 (1.00; 1.53)	14%, 0.28
NOS risk of bias			NA*			NA*			NA*
High quality	3	1.23 (1.06; 1.44)	0%, 0.53	2	1.22 (1.04; 1.42)	0%, 0.73	1	2.02 (0.81; 5.03)	NA

*, p-value of subgroup differences; NA, Not Applicable.

#### Sensitivity Analysis

Influence analyses conducted using random-effects models showed that no single study had a significant effect on the overall estimate or the heterogeneity (**Figures 3A, B**). The Baujat plot showed that the study of Cassimos et al. contributed the most to heterogeneity but had a small effect on the overall estimate. The Gaussian Mixture Model revealed that Brasky et al., Cassimos et al., Gross et al., and Howie et al. were potential outliers (**Appendices 2A, B**). We performed a separate analysis after the removal of the studies with outlier results which showed that the risk of breast cancer remained higher in the group with a history of tonsillectomy (OR, 1.23; 95% CI: 1.05-1.44, I^2^= zero) (**Appendix 2**).

## Discussion

This is the first comprehensive systematic review and meta-analysis examining the relationship of prior tonsillectomy previously and breast cancer amongst females. This meta-analysis included 8 studies in total, and the results demonstrate that there was a significant correlation between tonsillectomy and future development of breast cancer amongst adult females. Patients who underwent tonsillectomy previously (n = 2843), and women, in particular, were more prone to develop breast cancer later in their lives (n = 2200).

Theories put forward to explain this association suggests viral infections as the key driver of mutations, leading up to cancers. Late age tonsillectomies have been proposed as a proxy for a delayed type of infection by the Epstein- Barr virus (EBV) (17). Moreover, human papillomavirus (HPV) DNA has also been detected in tonsillectomy specimens, implying a possible causation agent for head and neck cancers (18).

Quite a few studies conducted previously have investigated the association of tonsillectomy with cancers at various locations. Vineis et al. in a case-control study depicted a two-fold risk of lymphocytic leukemia with a tonsillectomy performed at 10 years of age (5). Liaw et al. portrayed an increased risk of Hodgkin’s lymphoma in a cohort of Swedish patients, as opposed to the general population (3). In addition to that, there have been studies with mixed results showing a possible relation with prostate cancer as well (2, 19).

Lubin et al. showed an increased risk of breast cancer diagnosed after 65 years of age in women with a history of tonsillectomy (14). Yasui et al., on the other hand, showed an increased risk of breast cancer with tonsillectomies performed at >15 years of age (15). Brasky et al. in their study suggested a possible association between a history of tonsillectomy and future risk of development of breast cancer in premenopausal women (16). He proposed tonsillectomy to be an indicator of chronic inflammation in childhood, with subsequent increased risk of carcinogenesis (20). Moreover, prostaglandin production due to increased COX-2 activity in the setting of inflammation is correlated with estrogen synthesis and in turn, breast cell proliferation, in an *in-vitro* setting (21). Finally, the removal of tonsils, responsible for serving an important immunosurveillance function may lead to compromised immunologic defenses against cancer cell proliferation (22).

This study provides a deeper insight into the relationship between tonsillectomy and developing breast cancer; it extends and confirms previous results. Some other strengths of our study include precise results as a culmination of a comprehensive review that has not been done so far. Moreover, a comprehensive investigation of heterogeneity and use of sensitivity analyses to demonstrate the robustness of our results futher strengthen our meta-analysis.

Our study is not without limitations. Some of the major limitations that need to be highlighted include the observational nature of the included studies and the risk of confounding bias. Moreover, adjustment was not available for all of the studies, and some individual studies carried a high risk of bias. Due to inadequate reporting by the studies, we were not able to assess the association between the average age at tonsillectomy and risk of BC. Finally, there was a lack of matching between cases and controls in some studies.

Our study underscores the need for frequent follow-ups and screening of tonsillectomy patients to assess for the risk of BC. The indications for tonsillectomy may need to be reconsidered, especially in those patients with pre-existing risk factors for BC. Large-scale studies with a robust design to reduce confounding bias are needed to confirm our findings. Mechanistic research is also needed to fully understand the pathogenesis of BC in tonsillectomy patients.

## Data Availability Statemenet

The raw data supporting the conclusions of this article will be made available by the authors, without undue reservation.

## Author Contributions

SK, AE, MO, HC, AK: Data collection, analysis, screening and scientific writing. AB, MA, KD, LS, OR, NS: Data collection, analysis, screening and scientific writing. AA, JS, SG: Study concept, design, and drafting of the manuscript. All authors contributed to the article and approved the submitted version. SK, AA, JS, SG: Study concept, design, and drafting of the manuscript.

## Conflict of Interests

Author MO was employed by IQVIA.

The remaining authors declare that the research was conducted in the absence of any commercial or financial relationships that could be construed as a potential conflict of interest.

## Publisher’s Note

All claims expressed in this article are solely those of the authors and do not necessarily represent those of their affiliated organizations, or those of the publisher, the editors and the reviewers. Any product that may be evaluated in this article, or claim that may be made by its manufacturer, is not guaranteed or endorsed by the publisher.
