# Supplementary material for: Prior Tonsillectomy and the Risk of Breast Cancer in Females: A Systematic Review and Meta-analysis
Source: Front Oncol. 2022 Jul 20;12:925596. doi: 10.3389/fonc.2022.925596 (PMC9350012; doi:10.3389/fonc.2022.925596)
Supplement: Supplementary file 1 [file DataSheet_1.docx]

| **ppendix 1.** Risk of bias | | | | | | | | | | | |
| --- | --- | --- | --- | --- | --- | --- | --- | --- | --- | --- | --- |
| New-castle Ottawa Scale (NOS) | | | | | | | | | | | |
| Study | Case-control studies | | | | | | | | | | |
|  | Selection | | | |  | Comparability |  | Exposure | | | Risk  of bias |
|  | Is the case  definition  adequate? | Representativeness  of the cases | Selection  of Controls | Definition of Controls |  | Comparability of cases and controls on the basis of the design or analysis |  | Ascertainment of exposure | Same method of ascertainment for cases and controls | Non-Response rate |  |
| Brasky et al., 2009 | * | * | * | * |  | ** |  | * | * |  | High quality |
| Cassimos et al., 1973 |  |  | * | * |  |  |  |  | * |  | Very high risk |
| Gross et al., 1965 | * |  |  | * |  |  |  |  | * |  | Very high risk |
| Howie et al., 1965 | * |  | * | * |  |  |  |  | * |  | High risk |
| Kessler et al., 1970 | * |  |  | * |  |  |  |  | * |  | Very high risk |
| Lubin et al., 1982 | * | * | * | * |  | * |  |  | * |  | High risk |
| Yasui et al., 2001 | * | * | * | * |  | ** |  |  | * |  | High quality |
|  | Cohort studies | | | | | | | | | | |
|  | Selection | | | |  | Comparability |  | Outcome | | | Risk of bias |
|  | Demonstration  that outcome  of interest  was not  present at  start of study | Representativeness  of the exposed cohort | Selection  of the  non-exposed cohort | Ascertainment  of exposure |  | Comparability of cohorts on the basis of the design or analysis |  | Assessment of outcome | Was follow-up long enough for outcomes to occur | Adequacy of follow up of cohorts |  |
| Sun et al. 2015 | * | * | * | * |  | * |  | * | * | * | High quality |

**Appendix 2.** Influence analysis

A) Baujat plot

B) Gosh diagnosis test cluster plots

Brasky et al., Cassimo et al., Gross et al., and Howie et al., were identidied as outliers upon Gaussian Mixture Model

Influence analysis (continued)

C) Forest plot after removal of the outliers

**Appendix 3.** Subgroup analysis

A) All studies included

A.1) Subgrouped by study design

A.2) Subgrouped by year of publication

A.3) Subgrouped by continent

A.4) Subgrouped by country

A.5) Subgrouped by the sample size

A.6) Subgrouped by risk of bias

A.7) Subgrouped by age at diagnosis

A.7.1) premenopausal

A.7.2) postmenopausal

B) Studies from the US only

B.1) Subgrouped by study design

B.2) Subgrouped by year of publication

B.3) Subgrouped by the sample size

C) Non-US Studies

C.1) Subgrouped by study design

C.2) Subgrouped by the year of publication

C.3) Subgrouped by continent

C.4) Subgrouped by the sample size

**Appendix 4.** Search strategy

| **eTable 1.Search Queries** | | | | | | |
| --- | --- | --- | --- | --- | --- | --- |
|  | | | | | | |
|  | **Pubmed** | | | | | |
|  |  | 1. Exposure; tonsillectomy or tonsillitis ("Tonsillectomy"[Mesh] OR "Tonsillectomy" [tiab] OR "Tonsillectomies" [tiab] OR “Adenotonsillectomy" [tiab] OR "Adenotonsillectomies" [tiab] OR (tonsil[tiab] AND ablation[tiab])) | | | | |
|  |  | 2. Outcome ("Neoplasms"[Mesh] OR "Neoplasm"[tiab] OR "Neoplasms"[tiab] OR "Cancer"[tiab] OR "cancers"[tiab] OR "Tumors"[tiab] OR "Tumor"[tiab] OR "carcinoma"[tiab] OR "carcinomas"[tiab]) | | | | |
|  |  |  | | | | |
|  | **Embase** | |  |  |  |  |
|  |  | 1. Exposure; tonsillectomy or tonsillitis ('Tonsillectomy'/exp OR Tonsillectomy:ab,ti OR Tonsillectomies:ab,ti OR Adenotonsillectomy:ab,ti OR Adenotonsillectomies:ab,ti) AND 'human'/de AND 'article'/it | | | | |
|  |  | 2. Outcome  ('neoplasm'/exp OR Cancer:ab,ti OR cancers:ab,ti OR Neoplasm:ab,ti OR Neoplasms:ab,ti OR Tumors:ab,ti OR Tumor:ab,ti OR Tumours:ab,ti OR Tumour:ab,ti OR carcinoma:ab,ti OR carcinomas:ab,ti) AND 'human'/de AND 'article'/it | | | | |

We modified the same search strategy to fit: Scopus, Web of Science and Google scholar.
